# Asymmetry of fusiform structure in autism spectrum disorder: trajectory and association with symptom severity

**DOI:** 10.1186/s13229-016-0089-5

**Published:** 2016-05-24

**Authors:** Chase C. Dougherty, David W. Evans, Gajendra J. Katuwal, Andrew M. Michael

**Affiliations:** Autism and Developmental Medicine Institute, Geisinger Health System, 120 Hamm Drive, Lewisburg, PA 17837 USA; Department of Psychology, Program in Neuroscience, Bucknell University, 701 Moore Avenue, Lewisburg, PA 17837 USA; Chester F. Carlson Center for Imaging Science, Rochester Institute of Technology, Rochester, NY 14623 USA; Institute for Advanced Application, Geisinger Health System, 100 N Academy Ave, Danville, PA 17822 USA

**Keywords:** Fusiform gyrus, Asymmetry, Autism spectrum disorder, Development, Structural imaging

## Abstract

**Background:**

While asymmetry in the fusiform gyrus (FFG) has been reported in functional and structural studies in typically developing controls (TDC), few studies have examined FFG asymmetry in autism spectrum disorder (ASD) subjects and those studies are limited by small sample sizes, and confounded by cognitive ability or handedness. No previous work has examined FFG surface area or cortical thickness asymmetry in ASD; nor do we understand the trajectory of FFG asymmetry over time. Finally, it is not known how FFG structural asymmetry relates to ASD symptom severity.

**Methods:**

In this study, we examined FFG volume, surface area, and cortical thickness asymmetry, as well as their cross-sectional trajectories in a large sample of right-handed males aged 7 to 25 years with 128 ASD and 127 TDC subjects using general linear models. In addition, we examined the relationship between FFG asymmetry and ASD severity using the Autism Diagnostic Observation Schedule (ADOS) and Gotham autism severity scores.

**Results:**

Findings revealed that while group differences were evident with mean leftward asymmetry in ASD and mean near symmetry in TDC volume and surface area, asymmetry for both groups existed on a spectrum encompassing leftward and rightward asymmetry. In ASD subjects, volume asymmetry was negatively associated with ADOS and autism severity score symptom measures, with a subset of rightward asymmetric patients being most severely affected. We also observed differential trajectory of surface area asymmetry: ASD subjects exhibited a change from leftward asymmetry toward symmetry from age 7 to 25, whereas TDCs exhibited the reverse trend with a change from near symmetry toward leftward symmetry over the observed age range.

**Conclusions:**

Abnormalities in FFG structural asymmetry are related to symptom severity in ASD and show differential developmental trajectory compared to TDC. This study is the first to note these findings. These results may have important implications for understanding the role of FFG asymmetry in ASD.

**Electronic supplementary material:**

The online version of this article (doi:10.1186/s13229-016-0089-5) contains supplementary material, which is available to authorized users.

## Background

One of the more interesting features of the human brain is its structural and functional lateralization or asymmetry. Asymmetry occurs when structural or functional brain features are unequally represented in homotopic regions. Brain asymmetry may have developed as a mechanism for regional specialization [1] and as a way to reduce inter-hemispheric transfer of information [2]. As a result, typical patterns of brain asymmetry may confer added efficiency in neural processing.

One region which has shown asymmetry in both structure and function is the fusiform gyrus (FFG). The FFG is located in the inferior temporal lobe and contains a region referred to as the fusiform face area or FFA which is implicated in the processing and perception of faces [3]. Functionally, the left and right FFG are thought to exhibit differential roles, with the right FFG performing conscious processing of faces and the left FFG engaging in more general visual perception [4] and object recognition [5]. Functional imaging studies have reported that the right FFG exhibits preferential activation for faces compared to objects [6] and for unfamiliar vs. familiar faces [7]. Many patients suffering from prosopagnosia—the inability to identify faces—exhibit lesions in the right posterior FFG [8]. Consistent with this finding functional studies have largely shown that individuals exhibit a right hemisphere advantage when identifying whole faces, rather than parts [4]. In addition to its proposed role in face processing, research has also indicated that the left FFG [9] contains the visual word form area which is thought to be specialized for word recognition [10] and thus plays a critical role in reading, with lesions in this region resulting in alexia [11].

In autism spectrum disorder (ASD), impairments in face processing have been reported [12–15]. For example, patients with ASD have been shown to exhibit deficits in face memory [16] and in recognition of emotion in faces [17]. Abnormalities in circuitry encompassing the FFG and other regions such as the amygdala have been suggested to be a key component of impairment in ASD [15]. Thus FFG structure, and asymmetry specifically, may play a role in ASD. As a result, studying the link between FFG asymmetry and ASD impairment will shed further light on its potential role in the pathogenesis of ASD.

Relative to functional studies, there is a paucity of structural studies of the FFG, with some reporting leftward volumetric asymmetry (higher volume in left hemisphere) in controls in the posterior FFG [18, 19] or left FFG as a whole [1, 20, 21], although in Herbert et al. [1] FFG was not found to be significantly asymmetric as determined by a one sample *t* test. Findings of structural asymmetry may extend to the cellular level as well. A post-mortem study of FFG cytoarchitecture reported narrower mini-columns and fewer pyramidal neurons in the right, relative to the left FFG in healthy controls [22].

Lateralization is a normal phenomenon in typical brain development and there has been interest in examining possible disruption of typical patterns of brain lateralization in neurodevelopmental disorders [23]. In ASD in particular, deviations from typical patterns of asymmetry have been reported in both functional [24] and structural studies [1, 19, 25–27]. While structural asymmetry has been examined in ASD, only a few studies have examined asymmetry of the FFG with several studies indicating increased leftward volumetric asymmetry in posterior temporal FFG compared to controls [19] or leftward asymmetry (although not statistically different from zero asymmetry) in anterior and posterior FFG [1]. Further, histopathological studies of the FFG in ASD have reported structural abnormalities at the cellular level including reductions in neuron density, total neuron number, and perikaryal volume throughout the cortical layers of the FFG [28] as well as a decrease in GABA_B_ receptor density compared to controls [29].

Of the previous work examining FFG asymmetry, Herbert et al. [1, 19] included only 16 subjects with ASD over a narrow age range (7 to 11 years and 5.7 to 11.3 years, respectively). While both studies matched groups for handedness, cohorts in both studies included left-handed and right-handed subjects which may have introduced confounds as handedness is associated with brain lateralization [30]. Additionally, neither study appears to have controlled for IQ effects by matching groups or regressing IQ. Further, the previous work investigated the asymmetry of only the volume of the FFG. Research examining asymmetries in cortical thickness and surface area of the FFG are even more sparse, and to our knowledge, no studies have examined either morphometric feature in ASD. Cortical thickness and surface area are of interest as recent studies have found evidence that these may be dissociable features controlled by independent genetic mechanisms [31]. The volume of a cortical region is the product of its cortical thickness and surface area and as a result examining volume alone may not capture which of these measures influence observed volumetric asymmetries. Finally, it is not known if the developmental trajectory of FFG asymmetry in ASD differs from typical development.

For these reasons, we sought to examine volume, cortical thickness, and surface area asymmetries of the FFG and their trajectory in ASD using a large cross-sectional structural magnetic resonance imaging (sMRI) dataset. It should be noted that studies have indicated that subsections of the FFG have been associated with different functions. The FFA is thought to be located in the middle FFG [3] and its posterior aspects [32]. In contrast, the anterior FFG may play a role in semantic memory [33]. However, there are currently no clear anatomical or cytoarchitectonic criteria for identification of the FFA within the FFG [28]. The goal of this study is to examine the role of FFG asymmetry in the overall impairment of ASD and not domain specific-traits related to face processing or social impairment.

In this study, we examine a large homogeneous sample of right-handed males consisting of 128 high-functioning ASD subjects and 127 typically developing controls (TDC) across a large age range (7 to 25 years). In comparison to previous work examining FFG structural asymmetry [1, 19], our study consists of a large number of homogeneous subjects across a wider age range. This larger cohort affords greater statistical power in assessing group differences and allows for characterization of the trajectory of asymmetry in the FFG from mid-childhood to early adulthood. Here too, to our knowledge, no previous research has examined the development or trajectory of FFG asymmetry in ASD. Because the FFG is of particular relevance to ASD, we sought to examine how structural asymmetry may be related to impairment in this disorder. Thus, we also examined the relationship between degree of structural asymmetry in volume, surface area, and cortical thickness to ASD symptom severity using two different measures: ADOS (Autism Diagnostic Observation Schedule) [34] and Gotham autism severity scores [35].

## Methods

### Subjects

Data included in this study were obtained from the publicly available autism brain imaging data exchange (ABIDE) dataset which includes over 1100 sMRIs from 17 different sites [36]. Data quality of images was confirmed through visual inspection of each image. Images with severe motion (ghosting and smearing), susceptibility, and homogeneity artifacts were removed from further analysis. Images without complete head coverage were also removed. Only images with high fidelity were included for further analyses. A total of 172 images were removed due to poor image quality and another 64 subjects were removed due to segmentation failure during FreeSurfer preprocessing.

To reduce effects of handedness and gender, only right-handed male subjects were included. Subjects with missing handedness information, or missing data on verbal IQ (VIQ), performance IQ (PIQ), or full-scale IQ (FSIQ) were excluded. Subjects over the age of 25 years were also excluded due to the sparsity of data beyond this age as this could influence trajectory results. The final sample consisted of 128 ASD participants and 127 age-matched TDCs (demographics are presented in Table 1). Of the 128 ASD subjects, we analyzed a subset of 69 ASD participants with ADOS data, and a subset of 28 ASD participants with Gotham Autism Severity Score data (see Table 1). The final sample was matched in terms of age, PIQ, sex (only males), and handedness (only right-handed). Only VIQ differed between groups (*P* = 0.03); as communication impairment is a core symptom of ASD, this difference was expected.

**Table 1:** Subject Demographics. Table 1 contains mean age, verbal IQ (VIQ), performance IQ (PIQ), ADOS scores, and autism severity scores as well as standard deviations for ASD and TDC

	Full sample			ADOS sub-sample	Autism severity score sub-sample
	ASD *n* = 128 (mean ± SD)	TDC *n* = 127 (mean ± SD)	two-sample *t* test	ASD *n* = 69 (mean ± SD)	ASD *n* = 28 (mean ± SD)
Age (years) (range)	15.5 ± 4.1	15.6 ± 3.8	*t*_254_ = 0.2/*p* = 0.8	16.3 ± 3.8	13.3 ± 2.4
	7.3–24.0	7.7–25.0		8.5–24.0	8.5–17.9
VIQ	106.8 ± 13.3	110.9 ± 16.5	*t*_254_ = *2.2/p* = *0.03* *	106.8 ± 14.1	103.5 ± 13.1
PIQ	103.4 ± 13.6	105.8 ± 16.7	*t*_254_ = 1.3/*p* = 0.2	105.6 ± 14.7	100.2 ± 13.5
ADOS				11.7 ± 3.5	N/A
Autism severity score				N/A	6.7 ± 2.4

Significant ASD vs. TDC group differences are in italics with asterisk

*N/A* indicates not applicable

### Structural MRI

After exclusion criteria and quality control explained in the previous section were applied, the final sample consisted of structural MRIs from 8 of the 17 ABIDE sites. Scanner parameters of the eight sites are presented in Additional file 1: Table S1. Information on sites, subject recruitment and inclusion criteria and other details can be found at the ABIDE release website [37].

### Image processing

SMRI images were preprocessed and segmented using the *recon-all* whole-brain automated segmentation pipeline of FreeSurfer v5.3.0 [38]. FreeSurfer default templates, Desikan-Killiany atlas [39] for cortical regions and the *aseg* atlas [38] for subcortical regions, were used for segmentation. Volume, surface area, and cortical thickness measures for the FFG were computed.

### Data analysis

The symmetry-index (SI) [40] was calculated for each of the following measures: volume, surface area, and cortical thickness using Eq. 1, where L and R stand for the left and right region measures respectively. In Eq. 1, the difference between the left and right region measures is divided by the average of both left and right measures and multiplied by 100 to obtain the SI as a percentage. Negative values indicate rightward asymmetry percentage and positive values indicate leftward asymmetry percentage. The SI index has the added advantage of removing any possible confounds due to scanner site that may result from differences in scanner parameters and settings. Because SI is calculated as a percentage within each subject, rather than as pure morphological measures, possible confounds due to scanner site are removed.

1 $$ Symmetry\  Index\ (SI)=100\kern0.37em \times \frac{\left(L-R\right)}{\left(L+R\right)/2} $$

In preparation for general linear model (GLM) analysis, outliers were removed independently for each morphological index (volume, surface area, thickness). Subjects with measures that were two standard deviations above or below the combined group mean for each measure were excluded from the GLM analysis. This resulted in a sample size of 125/120 for volume, 127/121 for surface area, and 121/120 for cortical thickness for ASD/TDC cohorts, respectively. We then calculated the mean and standard deviations for ASD and TDC separately using unadjusted raw SI measures and performed a two-sample *t* test between the groups.

To test for the main effects of diagnosis for FFG SI, we created a GLM with the following variables: age, diagnosis, VIQ, and PIQ. An age × diagnosis interaction term was also added to the GLM (see Eq. 2). For the FFG SI-ADOS analysis and autism severity score analysis, we created a similar model with diagnosis, and age × diagnosis terms removed and included a term for symptom severity (where symptom severity is either ADOS or autism severity score). This model is depicted in Eq. 3. GLM analysis was conducted using the “*regstats*” function in MATLAB R2013B.

2 $$ SI={\beta}_0+{\beta}_1 age+{\beta}_2 diagnosis+{\beta}_3VIQ+{\beta}_4PIQ+{\beta}_5\mathrm{age}\times \mathrm{diagnosis}+\upepsilon $$

3 $$ SI={\beta}_0+{\beta}_1 age+{\beta}_2VIQ+{\beta}_3PIQ+{\beta}_4\mathrm{Symptom}\ \mathrm{Severity} + \upepsilon $$

**Fig. 1:**
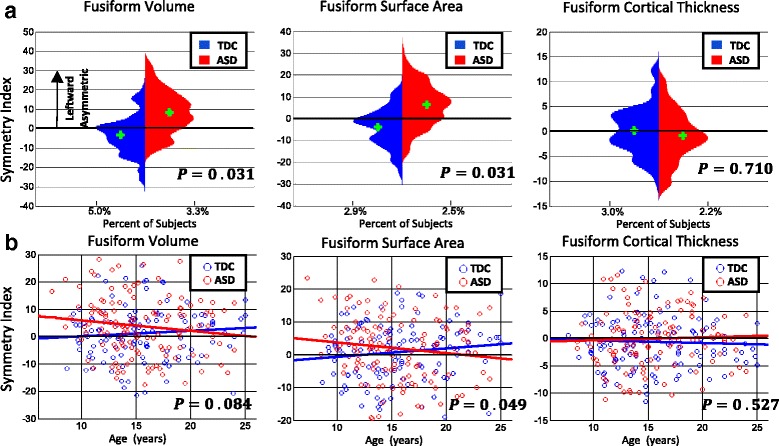
Histograms of symmetry index and age *×* diagnosis interaction plots. **a** Depicts histograms of symmetry index (adjusted for age and IQs) for ASD (*red*) and TDC (*blue*) for fusiform volume, surface area, and cortical thickness. Group means are presented by the *green crosshair*. Group mean differences were significant for volume *p* = 0.031 and surface area *p* = 0.031. **b** Depicts symmetry index across age range for cross-sectional data (adjusted for IQs). Age *×* diagnosis interaction was significant for surface area *p* = 0.049). Linear fit indicates a trend of decreasing leftward asymmetry with age toward symmetry for ASD and a change from nearly symmetric to leftward asymmetric for TDC

**Fig. 2:**
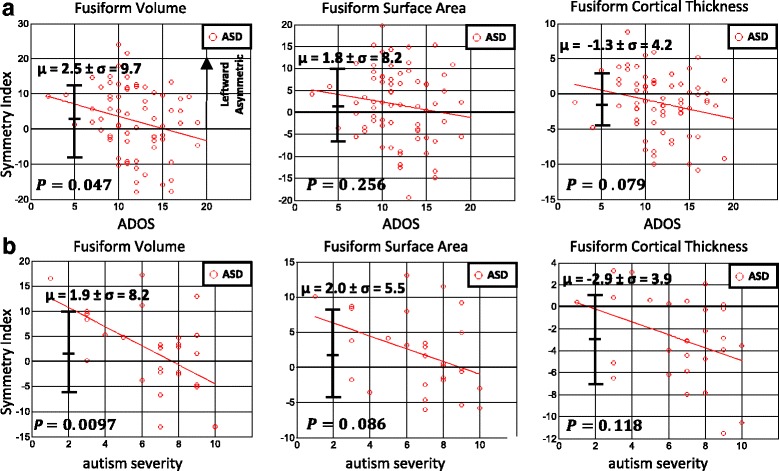
Symmetry index vs. ADOS and autism severity plots. **a** Depicts relationships between symmetry index and ADOS for fusiform volume, surface area, and cortical thickness in ASD. Results indicate a significant negative relationship between volume symmetry index and ADOS score (*p* = 0.047). **b** Depicts relationships between symmetry index and autism severity scores as measured by Gotham autism severity scores. A significant relationship between volume symmetry index and autism severity emerged (*p* = 0.0097). Mean and range of standard deviation are depicted for each plot. Data were adjusted for age and IQs

To visualize results from each of our GLM analyses via scatterplots and histograms, data were adjusted using Eq. 4 below; where *β*_*variable*_ is the beta coefficient from the corresponding GLM for the variable of interest, × _*variable*_ is the subject’s demographic data point for the variable of interest, and ${\overline{\times}}_{variable}$ is the sample mean for the demographic variable of interest. Removal of the effect of each additional covariate was performed by subtracting *β*_***variable***_$\left({\times}_{\boldsymbol{variable}}-{\overline{\times}}_{\boldsymbol{variable}}\right)$ from the subject’s SI measure for that covariate of interest. This was done for all subjects until a new set of adjusted SI values were obtained. Data in scatterplots depicting SI vs. age relationships (in Fig. 1a) are adjusted for VIQ and PIQ. Data in scatterplots depicting SI vs. ADOS and autism severity scores (in Fig. 2a and 2b) are adjusted for VIQ, PIQ, and age. After adjusted data points were plotted, a least squares line was fit to determine the directionality of relationships.

4 $$ S{I}_{adjusted}=SI-{\beta}_{age}\;\left(\mathrm{Age}-\overline{\mathrm{Age}}\right)-{\beta}_{VIQ}\left(\mathrm{V}\mathrm{I}\mathrm{Q}-\overline{\mathrm{VIQ}}\right)-{\beta}_{PIQ}\;\left(\mathrm{P}\mathrm{I}\mathrm{Q}-\overline{\mathrm{PIQ}}\right) $$

Using the same method, histogram plots of data adjusted for age, VIQ, and PIQ were created to illustrate SI distribution and group differences. Using a histogram allows the full distribution of SI to be visualized in addition to capturing the frequency of subjects at various degrees of SI.

Finally, in order to examine the effects of global measures on FFG SI, we compute pair-wise correlations between total brain, total brain SI, and FFG SI for volume, surface area, and thickness after performing outlier removal for each of these measures independently as described above.

## Results

### Unadjusted ASD vs. TDC SI differences

Mean **±** standard deviation for unadjusted raw volume, surface area, and cortical thickness SI for ASD/TDC, respectively, were as follows: volume SI (3.9 ± 11.0/1.2 ± 9.9), surface area SI (1.9 ± 9.8/0.74 ± 8.6), and cortical thickness SI (0.0055 ± 5.2/−0.74 ± 4.8). A two-sample *t* test of raw unadjusted SI revealed significant group differences for ASD vs. TDC for volume SI (*t*_243_ = − 2.01; *P* = 0.045). No significant group differences emerged for surface area SI or cortical thickness SI.

### Fusiform SI analysis

Results for the regression model for FFG volume, surface area, and cortical thickness SI are depicted in Table 2 (See Eq. 2 for modeling scheme). For measures with a significant main effect of diagnosis, the mean symmetry direction for ASD and TDC is indicated by L (leftward symmetric) or by S (nearly symmetric). Significant main effects of diagnosis were found for both FFG volume (*p* = 0.031) and FFG surface area (*p* = 0.031). The mean direction of asymmetry was leftward for both volume and surface area in ASD while TDC were nearly symmetric. Histograms of main effects for diagnosis are presented in Fig. 1a.

**Table 2:** Full Sample Fusiform SI Analysis *p*-values (*p* < 0.05). Table 2 contains significant values for the generalized linear model analysis

SI Measure	Age	Diagnosis (ASD/TDC)	VIQ	PIQ	Age *×* diagnosis
Fusiform volume	0.128	*0.031 (L/S)*	0.540	0.873	0.084
Fusiform surface area	0.137	*0.031 (L/S)*	0.787	0.668	*0.049*
Fusiform cortical thickness	0.656	0.701	0.097	0.854	0.527

For diagnosis, L indicates leftward asymmetry of mean SI while S indicates SI near zero (symmetric) for ASD/TDC, respectively

Significant relationships are indicated in italics

For all three SI measures (volume, surface area, and thickness), TDC are more symmetric on average than ASD participants. The maximum number of subjects in the histograms occurred at an SI of 5 % for ASD and 0 for TDC in volume asymmetry and 9 % for ASD and 0 for TDC in surface area asymmetry. Cortical thickness SI had a peak at −2 % for ASD and two peaks in TDC at 3 and −2 %. For volume asymmetry in ASD 28 subjects were rightward and 97 leftward asymmetric and for TDC 76 were rightward and 44 leftward asymmetric. For surface area asymmetry 35 ASD subjects were rightward and 92 leftward asymmetric and in TDC 82 were rightward and 39 leftward. In cortical thickness, 71 ASD subjects were rightward and 50 leftward and in TDC 62 subjects were rightward and 58 leftward. While group differences emerged in volume and surface area SI, histograms indicate a large degree of overlap in SI distributions between ASD and TDC with some ASD and TDC exhibiting opposite asymmetries in comparison to their respective group mean. Analyses also revealed that TDC mean for volume, surface area, and thickness was more symmetric on average (closer to zero) than in ASD.

A significant age × diagnosis interaction was found for FFG surface area (*p* = 0.049). In ASD, leftward surface area asymmetry decreased with age toward symmetry while in TDC a change from near symmetry to leftward asymmetry for surface area was found. This relationship held for volume as well although results only approached significance (*p* = .084*)*. For ASD surface area SI was approximately 5 at age 7 and −1 % at age 25 on average. For TDC surface area SI was nearly symmetric at ~−1 % at age 7 and 4 % at age 25 on average. Interactions for age × diagnosis relationships are depicted in Fig. 1b. Volume and surface area SI trajectories exhibited opposite trends for ASD and TDC with a loss of leftward asymmetry trending toward symmetry with age in ASD and a change from mostly symmetric toward leftward asymmetry with age for TDC. Cortical thickness, in contrast, appeared relatively stable across the observed age range for both groups. No significant relationships with VIQ or PIQ emerged, although the relationship between cortical thickness and VIQ approached significance (*p* = 0.097). *p* values are summarized in Table 2.

### ADOS and autism severity score sub-samples analysis

Results for the regression model for the ADOS and autism severity scores subsamples (see Eq. 3 for modeling scheme) are depicted in Table 3 below. The direction of relationships with symmetry index for significant regions (*p* < 0.05) is indicated by a positive or negative sign. In the ADOS sub-sample volume, SI was positively correlated with PIQ (*p* = 0.036) and negatively with ADOS (*p* = 0.047). FFG cortical thickness SI was also positively correlated with PIQ (*p* = 0.015) and negatively correlated with VIQ (*p* = 0.034).

**Table 3:** ADOS and Severity Score subsample *p*-values (*p* < 0.05): Table 3 contains significant values for the generalized linear model analysis for ADOS and severity scores sub-samples

	ADOS sub-sample				Severity scores sub-sample			
SI Measure	Age	VIQ	PIQ	ADOS	Age	VIQ	PIQ	Severity score
Fusiform volume	0.283	0.401	*0.036 (+)*	*0.047 (–)*	*0.011 (–)*	0.498	0.858	*0.0097 (–)*
Fusiform surface area	0.229	0.657	0.273	0.256	*0.009 (–)*	0.059	0.253	0.086
Fusiform cortical thickness	0.200	*0.034 (–)*	*0.015 (+)*	0.079	0.325	0.866	0.242	0.118

For significant main effects of age, VIQ, and PIQ, the direction of the relationship is indicated by a + or – sign

Significant values are in italics

Analysis of the severity scores revealed significant main effects of age for FFG volume (*p* = 0.011) and surface area SI (*p* = 0.009) as well as a significant relationship between FFG volume SI and severity scores (*p* = 0.0097). IQ measures were not significant in this sample (See Table 1).

FFG SI measures within both ADOS and autism severity scores sub-samples demonstrated negative associations with ASD symptom measures, although significance for this relationship only emerged in volume SI. These relationships are depicted in Fig. 2a for the ADOS sub-sample and Fig. 2b for the autism severity scores sub-sample. Means of the samples and their respective standard deviations are depicted as well.

### Raw total brain measures, total brain SI, and fusiform SI

Significant correlations were found between total brain volume SI and FFG volume SI (*r* = 0.27, *p* = 0.0039) and total brain surface area SI and FFG surface area SI (*r* = 0.22, *p* = 0.015) in TDC but these correlations were not significant in ASD. However, FFG cortical thickness SI was significantly correlated with both whole brain mean cortical thickness (*r* = −0.26, *p* = 0.0047) and whole brain mean cortical thickness SI (*r* = 0.33, *p* = 0.0003) in ASD but these correlations were not significant in TDC. Note that the FFG cortical thickness SI has a negative correlation with whole brain mean cortical thickness in ASD. All pair-wise correlations of this analysis are depicted in Additional file 2: Table S2.

## Discussion

Despite the abundance of literature examining the role of the FFG in ASD, few studies have examined FFG structural asymmetry in ASD. The little research that does exist indicates greater leftward volumetric asymmetry in posterior temporal FFG in ASD compared to TDC (20 vs. 6 %, respectively) [19], and leftward asymmetry in anterior and posterior FFG in both ASD and controls, although neither finding was significantly asymmetric after a one-sample *t* test [1]. Here, we report group differences in volume asymmetries in ASD and TDC, with nearly symmetric mean SI in TDC and leftward asymmetry in ASD (see Fig. 1a). While we did not parcellate the FFG into anterior and posterior sub-regions, our finding of leftward volume asymmetry in the FFG as a whole is consistent with Herbert et al.’s [1] report of leftward asymmetry in anterior and posterior FFG in ASD. For TDC, our findings indicated near symmetry in volume. Herbert et al. [19] reported leftward asymmetry in posterior FFG (6 %) and in a follow-up study [1] leftward asymmetry in anterior (17.40 %) and posterior FFG (6.20 %) in healthy children although these findings were not significantly asymmetric after a one-sample *t* test. Thus, while the direction of mean volume SI magnitude in TDC differed between our studies, our findings do not necessarily conflict. Overall, our findings indicate TDC on average appear to possess more symmetric FFG than ASD. The difference in average SI magnitude between our results and those of Herbert et al. may be attributed to differences in sample size, as well as IQ differences between cohorts. Here, we reported on a large sample of well-characterized ASD participants and accounted for VIQ, and PIQ as covariates in our GLM analysis. In the following sections, we discuss results which to our knowledge, have not been reported previously in ASD.

### Fusiform asymmetry trajectory

The results of our study indicate that asymmetry in surface area may not be constant throughout development. For surface area, this manifested as a change from leftward asymmetry toward symmetry with age in ASD and a change from initial symmetry to leftward asymmetry in TDC with age between early middle childhood and early adulthood. Whether these changes in asymmetry are driven by genetic mechanisms, environmental factors, or both is not known. However, previous work has demonstrated that structural asymmetry exists even prenatally [41, 42]. Post-mortem histological analyses have found evidence for genetic influence on brain lateralization in the form of differential gene expression in homotopic brain regions [43]. Brain regions which have been shown previously to exhibit lateralization were sampled, and evidence indicated that genes involved in nervous system development, glutamate receptor activity, and synaptic transmission exhibited lateralization in expression [43]. It is not entirely clear what factors influence asymmetry development. We speculate the observed aberrant trajectory in ASD could be the result of genetics or compensatory mechanisms/behavioral abnormalities related to impaired face processing. This is an important area for future research and merits further longitudinal analysis.

### Asymmetry and autism symptom severity

Both ADOS and autism severity scores (a standardized ADOS score) were inversely associated with volume SI in ASD. That is, ASD subjects exhibit greater rightward FFG asymmetry with increasing symptom severity. It appears that while ASD is on average leftward asymmetric (See Fig. 2a, b), there exists a subset of more severely affected subjects who exhibit rightward asymmetry and who are atypical compared to the majority of the ASD population. From the results of our full-sample analysis, only 28 subjects with ASD exhibited rightward asymmetry while 97 exhibited leftward. This is consistent with recent research indicating that ASD results from a variety of genetic etiologies [44]. This subset of more severely affected individuals with rightward volumetric asymmetry may represent a distinct subtype or etiology. Higher functioning subjects with ASD may therefore be more leftward asymmetric in volume while lower functioning individuals may be more rightward asymmetric. Similar relationships between ASD symptom measures and surface area SI and cortical thickness SI were found, although neither was significant. No previous work has examined the relationship of structural asymmetry in the FFG to autism symptom measures. Although some functional studies have suggested that the FFA does not play a critical role in reported face processing abnormalities and social impairment [45, 46], our findings at least lend support to the idea that structural asymmetries in the FFG are related to symptom severity in ASD. Such findings pave the way for research examining both etiological and symptom severity threshold effects pertaining to FFG asymmetry, as well as considering the relative volume of FFG to other structures.

### Asymmetry and IQ

FFG SI was associated with IQ only in analyses of the sample with ADOS scores, where FFG volume correlated with VIQ, and cortical thickness with VIQ and PIQ. This result did not emerge in analyses pertaining to the Gotham autism severity scores, which may be due to the small number of subjects in those analyses (*n* = 28). The lack of significant IQ relationships in our full-sample may indicate that asymmetry is only a significant predictor for VIQ and PIQ in ASD. In addition, in cortical thickness SI, the directionality of the relationships for VIQ and PIQ differed: SI was negatively associated with VIQ, and positively for PIQ. Thus VIQ was associated with greater rightward asymmetry and PIQ was associated with greater leftward asymmetry. Given that PIQ measures processing speed and perceptual ability and VIQ measures assess verbal skill and working memory, these opposing relationships indicate that measures of intellectual functioning may best be examined at the level of more basic cognitive constructs rather than full IQ measures.

### Relationship between volume, surface area, and cortical thickness

From our results, it appears that cortical thickness asymmetry exhibits the least difference between ASD and TDC. Results of the full sample GLM analysis indicated no effect of diagnosis nor any differences in asymmetry trajectory. It appears then that cortical thickness asymmetry is a more stable feature than volume or surface area asymmetry across the observed age range (see Fig. 1b). Of the three asymmetry measures examined, only cortical thickness SI failed to differentiate diagnostic groups, or to reveal developmental change over time. This suggests that differences in volume asymmetry trajectory are likely driven by changes in surface area rather than cortical thickness. Our results are consistent with reports that surface area and cortical thickness are independent brain features controlled by separate genetic mechanisms [31]. For example, group differences were observed in FFG volume and surface area, but not cortical thickness for our full-sample analysis. Similarly, volume and cortical thickness exhibited significant relationships with IQ measures, but not surface area. This emphasizes the need to treat surface area and cortical thickness as independent measures with potentially different clinical, behavioral, and developmental implications. In addition, each measure may be reflective of differing cellular abnormalities. In ASD, previous work has indicated neuropathological alterations in cortex in the FFG including reductions in neuron density and number as well as mean perikaryal volume [28]. The findings reported here in FFG surface area may therefore be indicative of specific histopathological changes. Future structural imaging research in ASD should thus carefully consider both cortical thickness and surface area as separate components of gross volumetric changes.

### Effect of global brain measures on fusiform SI

Correlation among total brain measures, total brain SI measures, and FFG SI measures for volume, surface area, and cortical thickness revealed several findings (see Additional file 2: Table S2). Although the direction of overall brain volume SI was correlated positively with FFG volume SI in TDC, this was not the case in ASD and thus we can be sure that the observed relationship between FFG volume SI and autism severity in ASD is not due to global asymmetry in volume. Similarly, this relationship was also observed in total surface area SI and FFG surface area SI in TDC but not in ASD. The only global measures in ASD to exhibit effects on FFG SI were whole-brain mean cortical thickness and whole-brain mean cortical thickness SI. However, in our findings FFG cortical thickness SI was not significantly associated with autism severity. The above results provide evidence that the global measures did not contribute towards the FFG asymmetry and autism severity results we report in ASD.

### Limitations

This study has several limitations. First, we used a cross-sectional dataset and the age range of the sample was limited to 7–25 years. Further support for findings relating to asymmetry development would need to be confirmed using longitudinal datasets, across a wider age range. A longitudinal study would afford higher statistical power in capturing the course of individual asymmetry development. In addition, examining subjects before age 7 would give better insight into the development of asymmetry and whether asymmetry is present at a young age or emerges over time. This is especially important given the consistent findings that reveal important morphological (i.e., head circumference) changes in ASD in early development [47, 48]. Furthermore, individuals diagnosed with ASD can span a continuum from non-verbal with intellectual disability to high-functioning individuals. The full sample has a mean FIQ of 105.8 for the ASD group and as such we cannot comment on asymmetry in lower functioning ASD populations. Next, the data were acquired from multiple sites with differing scanner parameters; however, using the symmetry index has two significant advantages: (1) the symmetry index inherently controls for total brain volume by dividing the difference between homotopic regions by their average and (2) because we used data acquired from the multi-site ABIDE data set, concerns over inter-site differences in scanner acquisition parameters are removed as well due to the fact the symmetry index is calculated as a percentage for each individual subject. In contrast, comparing pure morphological measures introduces confounds due to scanner and acquisition parameters. Finally, while the FFA is thought to be present in the middle and posterior FFG, there are no established structural landmarks with which to establish its location within the FFG [28]. Because we used measures which only measure general impairment in ASD, it is unclear how abnormalities in FFG asymmetry relate to impairments in face processing. This is an important area for future research to explore. In the future, it will also be critically important to better characterize the ASD sample from a genetics-first approach. There are hundreds of genetic mutations that confer risk for ASD, including whole chromosome aneuploidies, copy number variation syndromes, single gene mutations, and single nucleotide polymorphisms and variants. The genomic changes unquestionably have differential effects on symptom severity, developmental course, and brain morphology.

## Conclusions

In this study, we examined asymmetries in volume, surface area, and cortical thickness in the FFG in a population of individuals diagnosed with ASD and controls (TDC). We report significant group differences in FFG volume and surface area asymmetries between individuals with ASD and matched TDCs with TDC being nearly symmetric and ASD exhibiting mean leftward asymmetry. While differences in group means emerged, asymmetry was widely distributed for both groups with some subjects exhibiting opposite direction of asymmetry compared to their respective group mean. In addition, findings indicate that these asymmetries may change over time. We report that asymmetry in surface area exhibits differential development in ASD compared to TDC. ASD patients exhibit decreasing leftward asymmetry progressing toward symmetry with age and controls show leftward asymmetry with age while initially exhibiting an SI close to zero. Whether asymmetry changes beyond the age range observed in either ASD or TDC is uncertain, however. Furthermore, findings indicate that volume asymmetry in ASD exhibits a significant relationship with autism symptomatology as measured by the ADOS and autism severity scores. These findings may have important implications for impairment in ASD. In the future, research should further examine structural asymmetry in the FFG to determine its relation to specific domains of dysfunction in ASD.

### Ethics approval and consent to participate

This study was approved by the Institutional Review Board at Geisinger Health System and made use of the publically available ABIDE dataset. Consent to participate was obtained via IRB approval or explicit waiver at participating ABIDE sites. All data were fully anonymized in accordance with HIPAA guidelines. For more information see Di Martino et al. 2010 [36].

### Consent for publication

Not applicable.

### Availability of data and materials

The dataset supporting the conclusions of this article is available in the Autism Brain Imaging Data Exchange repository [http://fcon_1000.projects.nitrc.org/indi/abide/].

## Additional files

Additional file 1: Table S1. Scanner Parameters. (DOCX 12 kb) Additional file 2: Table S2. Correlations for Total Brain, Total Brain SI, and Fusiform SI (p < .05): Correlations between total brain, total brain SI and fusiform SI are presented for volume, surface area, and mean cortical thickness. Bolded values indicate significant relationships. Left and right brain volume totals for total brain volume SI calculation were computed by adding left and right cortical and cerebellar grey and white matter from Freesurfer in addition to all subcortical outputs for which a left and right measures were provided. (DOCX 12 kb)

## Abbreviations

ABIDE: autism brain imaging data exchange
ADOS: autism diagnostic observation schedule
ASD: autism spectrum disorder
FFA: fusiform face area
FFG: fusiform gyrus
FSIQ: full-scale IQ
GLM: general linear model
PIQ: performance IQ
SI: symmetry index
sMRI: structural magnetic resonance imaging
TDC: typically developing control
VIQ: verbal IQ
