# Supplementary material for: Asymmetry of fusiform structure in autism spectrum disorder: trajectory and association with symptom severity
Source: Mol Autism. 2016 May 24;7:28. doi: 10.1186/s13229-016-0089-5 (PMC4879740; doi:10.1186/s13229-016-0089-5)
Supplement: Additional file 1: Table S1. — Scanner Parameters. (DOCX 12 kb) [file 13229_2016_89_MOESM1_ESM.docx]

| Additional file 1: Table S1 Scanner Parameters | | | | | | | | | |
| --- | --- | --- | --- | --- | --- | --- | --- | --- | --- |
| Site | **Scanner Model** | **Scan**  **Time (min)** | **Voxel**  **Size**  **(mm)** | **Orientation** | **Slices**  **Per**  **Slab** | **Slice**  **Thickness**  **(mm)** | **TR**  **(sec)** | **TE**  **(sec)** | **Echo**  **Spacing**  **(sec)** |
| CALTECH | SMTS | 3:43 | 1 | Sagittal | 176 | 1 | 1590 | 2.73 | 7.9 |
| CMU | SMVS | 4:21 | 1 | Sagittal | 176 | 1 | 1870 | 2.48 | 7.4 |
| KUL | PI |  | 0.97 | Coronal | 182 |  |  | 4.6 |  |
| PITT | SMAS | 8:59 | 1 | Sagittal | 176 | 1.05 | 2100 | 3.93 | 9.4 |
| SJH | PA | 7:29 | 1 | Sagittal | 160 |  | 8.5 | 3.9 |  |
| UCLA | SMTS | 9:14 | 1 | Sagittal | 160 | 1.2 | 2300 | 2.84 | 6.6 |
| UM | GS |  |  | Axial | 40 | 3 | 250 | 5.7 |  |
| YALE | SMTS | 8:34 | 1 | Sagittal | 176 | 1 | 1230 | 1.73 | 5.4 |
