# Supplementary material for: Asymmetry of fusiform structure in autism spectrum disorder: trajectory and association with symptom severity
Source: Mol Autism. 2016 May 24;7:28. doi: 10.1186/s13229-016-0089-5 (PMC4879740; doi:10.1186/s13229-016-0089-5)
Supplement: Additional file 2: Table S2. — Correlations for Total Brain, Total Brain SI, and Fusiform SI (p < .05): Correlations between total brain, total brain SI and fusiform SI are presented for volume, surface area, and mean cortical thickness. Bolded values indicate significant relationships. Left and right brain volume totals for total brain volume SI calculation were computed by adding left and right cortical and cerebellar grey and white matter from Freesurfer in addition to all subcortical outputs for which a left and right measures were provided. (DOCX 12 kb) [file 13229_2016_89_MOESM2_ESM.docx]

| **Additional file 2: Table S2 Correlations for Total Brain, Total Brain SI, and Fusiform SI** | | | | | | |
| --- | --- | --- | --- | --- | --- | --- |
|  | Volume | | Surface Area | | Cortical Thickness | |
| Correlation | ASD | TDC | ASD | TDC | ASD | TDC |
| total brain – total brain SI | r = 0.033  p = 0.73 | r = $-$0.012  p = 0.90 | r = 0.13  p = 0.17 | r = $-$0.062  p = 0.51 | r = $-$0.14  p = 0.14 | r = 0.14  p = 0.14 |
| total brain – fusiform SI | r = 0.067  p = 0.46 | r = 0.084  p = 0.37 | r = 0.080  p = 0.39 | r = 0.076  p = 0.42 | **r =** $-$**0.26**  **p = 0.0047** | r = $-$0.012  p = 0.90 |
| total brain SI – fusiform SI | r = 0.012  p = 0.90 | **r = 0.27**  **p = 0.0039** | r = $-$0.13  p = 0.16 | **r = 0.23**  **p = 0.015** | **r = 0.33**  **p = 0.0003** | r = 0.050  p = 0.59 |
